# MgCBP1 Synergistically Enhances MgENG2‐Mediated Cellulose Degradation to Promote Parasitism in *Meloidogyne graminicola*

**DOI:** 10.1111/mpp.70346

**Published:** 2026-09-17

**Authors:** Chunhui Huang, Yuqing Cao, Ao Xu, Jiani Chen, Borong Lin, Qiuling Huang, Kan Zhuo

**Affiliations:** ^1^ College of Plant Protection South China Agricultural University Guangzhou China

**Keywords:** β‐1,4‐endoglucanase, cellulose degradation, cellulose‐binding protein, *Meloidogyne graminicola*, synergism

## Abstract

Plant‐parasitic nematodes secrete effector proteins, including cell wall‐degrading enzymes (CWDEs) and cell wall‐modifying proteins (CWMPs), to facilitate infection. However, whether cellulose‐binding proteins (CBPs), which are a class of CWMPs, can synergize with cellulases (a subgroup of CWDEs) to promote parasitism remains unclear. Here, we identified and characterized a CBP (*MgCBP1*) and a β‐1,4‐endoglucanase (*MgENG2*) from the rice root‐knot nematode *Meloidogyne graminicola*. MgCBP1 contains a conserved cellulose‐binding module (CBM) and is specifically expressed in the subventral oesophageal glands, with transcript levels markedly up‐regulated in pre‐parasitic second‐stage juveniles (pre‐J2s) and during early infection. Recombinant MgCBP1 bound cellulose, disrupted fibre structure and enhanced cellulase‐mediated hydrolysis via its intact CBM, whereas a CBM‐disrupted mutant lack both activities. MgENG2, which lacks a CBM, exhibited strong endoglucanase activity and displayed an expression profile similar to that of *MgCBP1*. MgCBP1 significantly enhanced the hydrolytic efficiency of MgENG2, whereas its CBM‐disrupted mutant did not. Transgenic rice overexpressing *MgCBP1* showed enhanced susceptibility to *M. graminicola* . RNAi‐mediated individual silencing of *MgCBP1* or *MgENG2* both reduced infection, with *MgENG2* silencing showing a significantly greater effect. Co‐silencing of both genes yielded the greatest reduction in gall formation and parasitic nematode numbers, which was significantly greater than that of *MgCBP1*‐only silencing, but did not differ significantly from *MgENG2*‐only silencing. Collectively, these findings demonstrate that MgCBP1 promotes nematode parasitism by binding cellulose, disrupting fibril surface integrity and enhancing the cellulolytic efficiency of MgENG2, providing the first experimental evidence that a CBP from a plant pathogen can synergistically facilitate infection by boosting endoglucanase‐mediated cellulose degradation.

## Graphical abstract

A cellulose‐binding protein (MgCBP1) and a β‐1,4‐endoglucanase (MgENG2) from *Meloidogyne graminicola* act synergistically. MgCBP1 binds cellulose, disrupts fibre structure and enhances MgENG2‐mediated cellulose degradation via its cellulose‐binding module. This cooperative action promotes nematode infection, providing the first evidence of CBP–cellulase synergy in a plant pathogen.

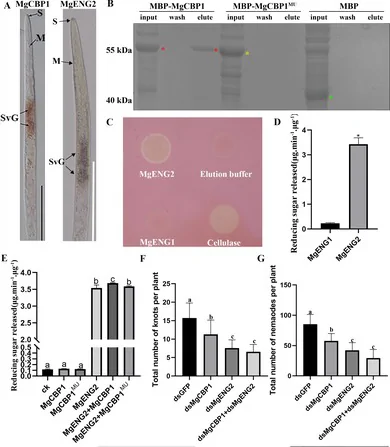

## 1 Introduction

Plants are attacked and parasitized by various pathogens, including fungi, bacteria and nematodes, during their growth and development. These pathogens secrete effectors to facilitate invasion and establish parasitic relationships (Mejias et al. 2021; Zhao et al. 2024). Cell wall‐degrading enzymes (CWDEs), such as endoglucanases, ligninases and polygalacturonases, represent a major group of early‐infection effectors that degrade cellulose, lignin and pectin in plant cell walls, respectively (Eves‐Van Den Akker et al. 2021; Haegeman et al. 2012). Among these, β‐1,4‐endoglucanases are notable for their widespread presence in plant pathogens. In plant‐parasitic nematodes (PPNs), β‐1,4‐endoglucanase was the first effector identified (Smant et al. 1998), and has since been detected in a broad range of major PPNs, including root‐knot nematodes (RKNs), cyst nematodes (CNs), lesion nematodes and the pine wood nematode (Gao et al. 2004a; Hu et al. 2013; Uehara et al. 2001; Shibuya and Kikuchi 2008). Functional studies have demonstrated that these enzymes contribute to nematode parasitism. For example, silencing of a β‐1,4‐endoglucanase in *Meloidogyne javanica* and *Globodera rostochiensis* significantly reduced infection efficiency (Hu et al. 2013; Rehman et al. 2009).

Cellulose, a linear polymer of glucose units linked by β‐1,4‐glycosidic bonds, is the major structural component of plant cell walls (Brás et al. 2011). Accordingly, it was traditionally believed that nematodes secrete endoglucanases during early infection to hydrolyse these bonds, thereby degrading the cell wall to facilitate invasion and migration (Ledger et al. 2006; Long et al. 2013). However, recent studies have revealed that nematode β‐1,4‐endoglucanases perform additional functions beyond cell wall degradation. For instance, the β‐1,4‐endoglucanase PcENG2 from *Pratylenchus coffeae* targets and inhibits maize chitinase ZmCHI5, consequently attenuating host basal immunity (Wang et al. 2025). Another example, PcENG3, targets maize translationally controlled tumour protein ZmTCTP2a to suppress immune responses while also regulating root cell proliferation, differentiation and development, thereby promoting nematode parasitism (Wang et al. 2026). Additionally, HmENG1 from *Hirschmanniella mucronata* interacts with rice OsGAPDH, interfering with carbon metabolism and jasmonic acid‐related pathways (Liu et al. 2026).

In addition to effectors that directly degrade cell wall components, plant pathogens also secrete effectors involved in cell wall modification, such as expansins and cellulose‐binding proteins (CBPs). CBPs contain a cellulose‐binding module (CBM) but lack intrinsic cellulolytic activity; instead, they specifically bind to cellulose substrates through this module (Hall et al. 1995; Hervé et al. 2010). Such proteins have been identified in various PPNs, and their CBMs have been shown to exhibit typical cellulose‐binding activity. For example, MiCBP from *M. incognita* and HgCBP from *Heterodera glycines* both bind cellulose substrates (Ding et al. 1998; Gao et al. 2004b). Beyond this binding function, nematode CBPs also perform additional roles. For instance, HsCBP from *Heterodera schachtii* interacts with *Arabidopsis* pectin methylesterase AtPME3, modulating its activity to regulate cell elongation and thereby promoting parasitism (Hewezi et al. 2008). Notably, although the expansin BsEXLX1 from *Bacillus subtilis* has been shown to act synergistically with cellulase to enhance cellulose degradation (Kim et al. 2009), whether a similar synergistic relationship exists between CBPs and cellulases in plant pathogens remains unknown.

RKNs are among the most economically destructive PPNs, causing substantial yield losses to staple and cash crops worldwide. *Meloidogyne graminicola* , in particular, is a major threat to rice production (Rusinque et al. 2021). However, neither β‐1,4‐endoglucanases nor CBPs have previously been characterized in this species. Here, we identify and functionally characterize a CBP (MgCBP1) and a β‐1,4‐endoglucanase (MgENG2). Both genes are specifically expressed in the subventral oesophageal glands and show elevated transcript levels in pre‐parasitic and early parasitic stages. Mechanistically, MgCBP1 binds cellulose, disrupts fibril surface integrity and enhances the degradation efficiency of cellulases, while MgENG2, which lacks a CBM, exploits both the binding and surface‐disrupting activities of MgCBP1 to increase its own catalytic efficiency, thereby contributing to parasitism. Collectively, our findings provide the first experimental evidence that a CBP protein from a plant pathogen can act synergistically with an endoglucanase to promote cellulose degradation and facilitate infection.

## 2 Results

### 2.1 Sequence Analysis of *MgCBP1* From *M. graminicola*

**FIGURE 1:**
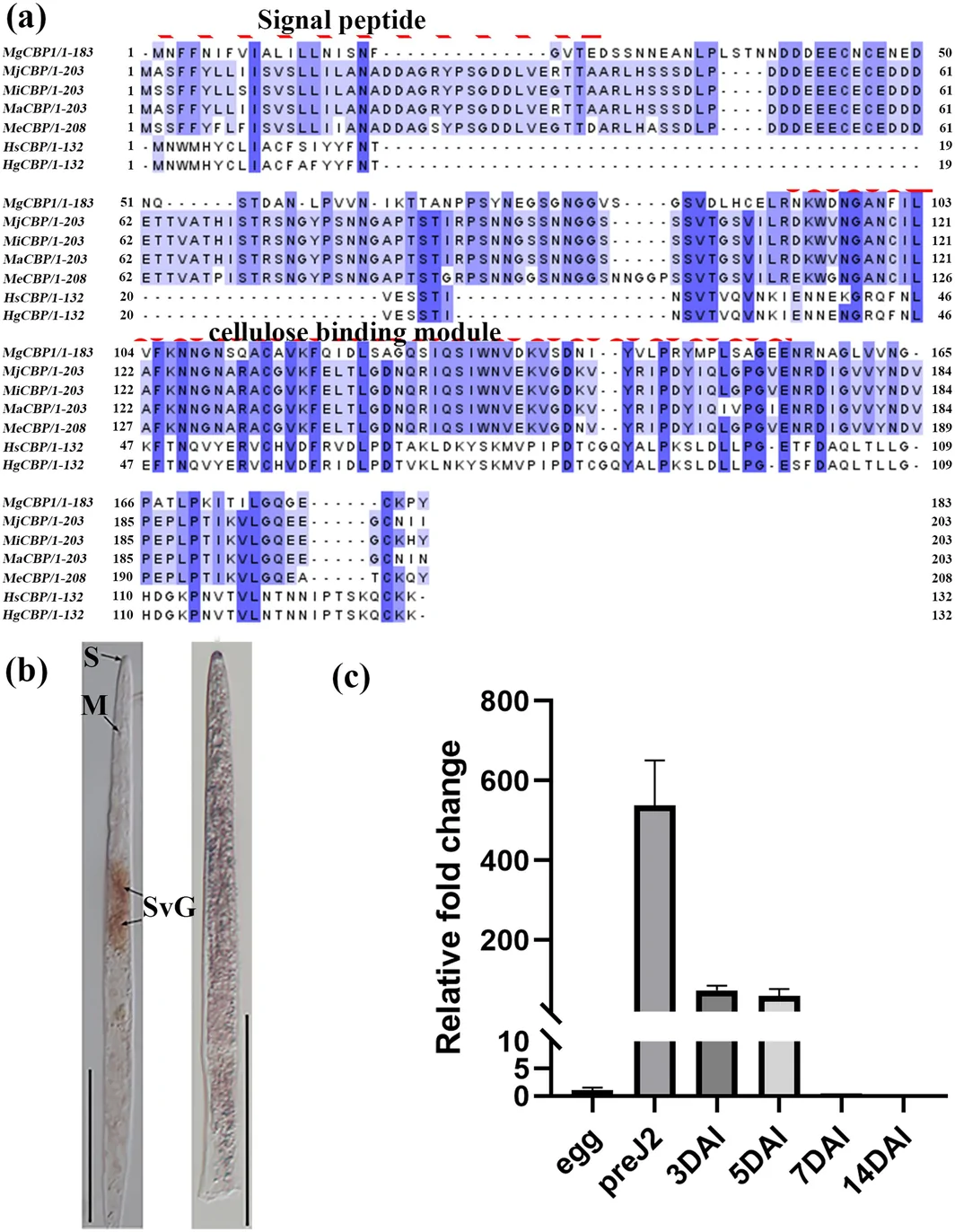
Sequence analysis and expression profile of MgCBP1 in *Meloidogyne graminicola*. (a) Multiple sequence alignment of MgCBP1 with homologous nematode cellulose‐binding protein. (b) In situ hybridization localization of MgCBP1 in the subventral gland cells of *M. graminicola* pre‐parasitic second‐stage juveniles (pre‐J2s). S, stylet; M, median bulb; SvG, subventral gland cells. Scale bar = 100 μm. (c) Development expression profile of *MgCBP1* across eggs, pre‐J2s and four post‐infection time points by reverse transcription‐quantitative PCR. The relative fold change values were calculated using the 2^−ΔΔ*Ct*^ method and normalized to the expression level in eggs. Data are presented as mean ± standard error of three independent biological replicates. DAI, days after inoculation.

Based on the genome sequence of *M. graminicola* (PRJNA411966), a CBP gene, designated *MgCBP1*, was amplified. The *MgCBP1* gene contains a 552‐bp open reading frame (ORF) interrupted by three introns of 59, 86 and 388 bp. The ORF encodes a predicted protein of 183 amino acids (aa), with a calculated molecular mass of 16 kDa. SignalP and TMHMM analyses predicted an N‐terminal signal peptide of 22 amino acids (aa), and no transmembrane domain was detected. InterPro domain analysis revealed a CBM spanning residues 92–154 (Figure 1a).

Multiple sequence alignment showed that the CBM of MgCBP1 is relatively conserved among orthologues from other RKN species, whereas the N‐terminal flanking regions are variable (Figure 1a). Pairwise identity analysis based on full‐length protein sequences revealed that MgCBP1 shares 52.2%–54.6% sequence identity with its RKN orthologs, including those from *M. incognita* (AF049139), *M. enterolobii* (KU350655), *M. arenaria* (CAM33389) and *M. javanica* (AM491771). In contrast, the overall sequence identity with CN proteins was lower, with only 30.4% to both *H. glycines* (AAN32887) and *H. schachtii* (ABY49997).

### 2.2 Spatiotemporal Expression of *MgCBP1* in *M. graminicola*

The tissue localization of *MgCBP1* was examined by in situ hybridization using a digoxigenin (DIG)‐labelled antisense probe in pre‐parasitic second‐stage juveniles (pre‐J2s) of *M. graminicola* . Strong hybridization signals were specifically detected in the subventral oesophageal glands, whereas no signal was observed in the negative control probed with the sense probe (Figure 1b).

The developmental expression profile of *MgCBP1* was further assessed by reverse transcription‐quantitative real‐time PCR (RT‐qPCR) across eggs, pre‐J2s, and four post‐infection time points. Relative transcript levels at each stage were calculated using the egg stage as the reference (set to 1.0). *MgCBP1* expression peaked at the pre‐J2 stage, with transcript abundance approximately 500‐fold higher than that in eggs. Elevated expression persisted at 3 days post‐infection (dpi) and 5 dpi, representing approximately 150‐ and 130‐fold increases relative to eggs, respectively, after which transcript levels dropped sharply (Figure 1c). Taken together, these results suggest that MgCBP1 may function as an effector involved in the invasion and early parasitic stages of *M. graminicola* .

### 2.3 MgCBP1‐Overexpressing Rice Exhibits Enhanced Susceptibility to *M. graminicola*

**FIGURE 2:**
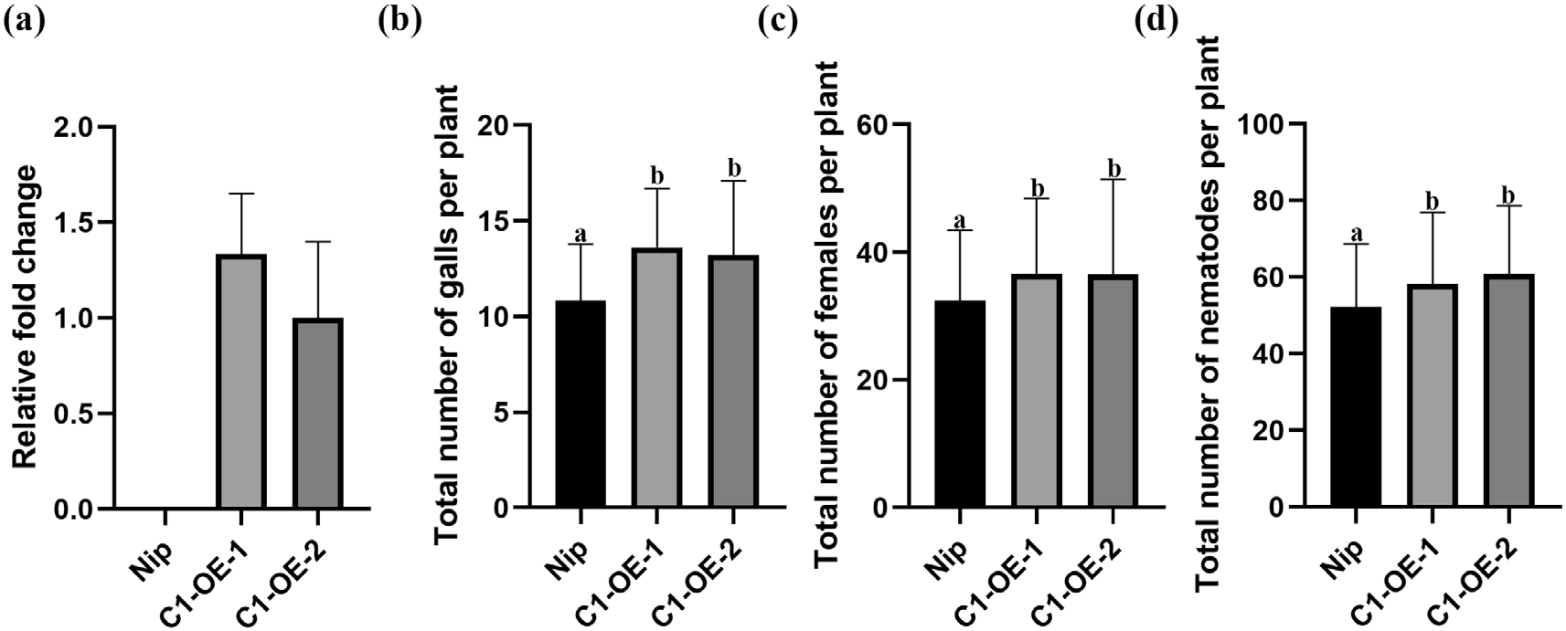
*MgCBP1*‐overexpressing rice exhibits enhanced susceptibility to *Meloidogyne graminicola*. (a) Relative expression levels of *MgCBP1* in roots of wild‐type (Nip) and *MgCBP1* overexpression (OE) transgenic lines, as determined by reverse transcription‐quantitative PCR. Transcript levels were normalized to those in line C1‐OE‐2, which was set to 1.0. (b–d) Infection assays showing the total number of galls (b), females (c) and nematodes (d) per plant. The transgenic OE lines exhibited significantly higher gall, female and nematode counts than the Nip control. Data are presented as the means ± standard error. Different letters above the bars indicate statistically significant differences (*p* < 0.05). Nip, wild‐type ( *Oryza sativa* ‘Nipponbare’); C1‐OE‐1 and C1‐OE‐2, independent T_2_ transgenic lines overexpressing *MgCBP1*.

To determine whether *MgCBP1* contributes to nematode parasitism, transgenic rice ( *Oryza sativa* ‘Nipponbare’) overexpressing *MgCBP1* was generated. Overexpression of *MgCBP1* in two independent transgenic lines (C1‐OE‐1 and C1‐OE‐2) was confirmed by RT‐qPCR (Figure 2a). In infection assays with *M. graminicola* , these overexpression lines exhibited significantly enhanced susceptibility to *M. graminicola* compared with wild‐type controls (Figure 2b–d). Collectively, these results suggest that MgCBP1 promotes *M. graminicola* parasitism.

### 2.4 MgCBP1 Binds Cellulose, Disrupts Fibre Structure and Promotes Cellulase‐Mediated Hydrolysis via Its CBM

**FIGURE 3:**
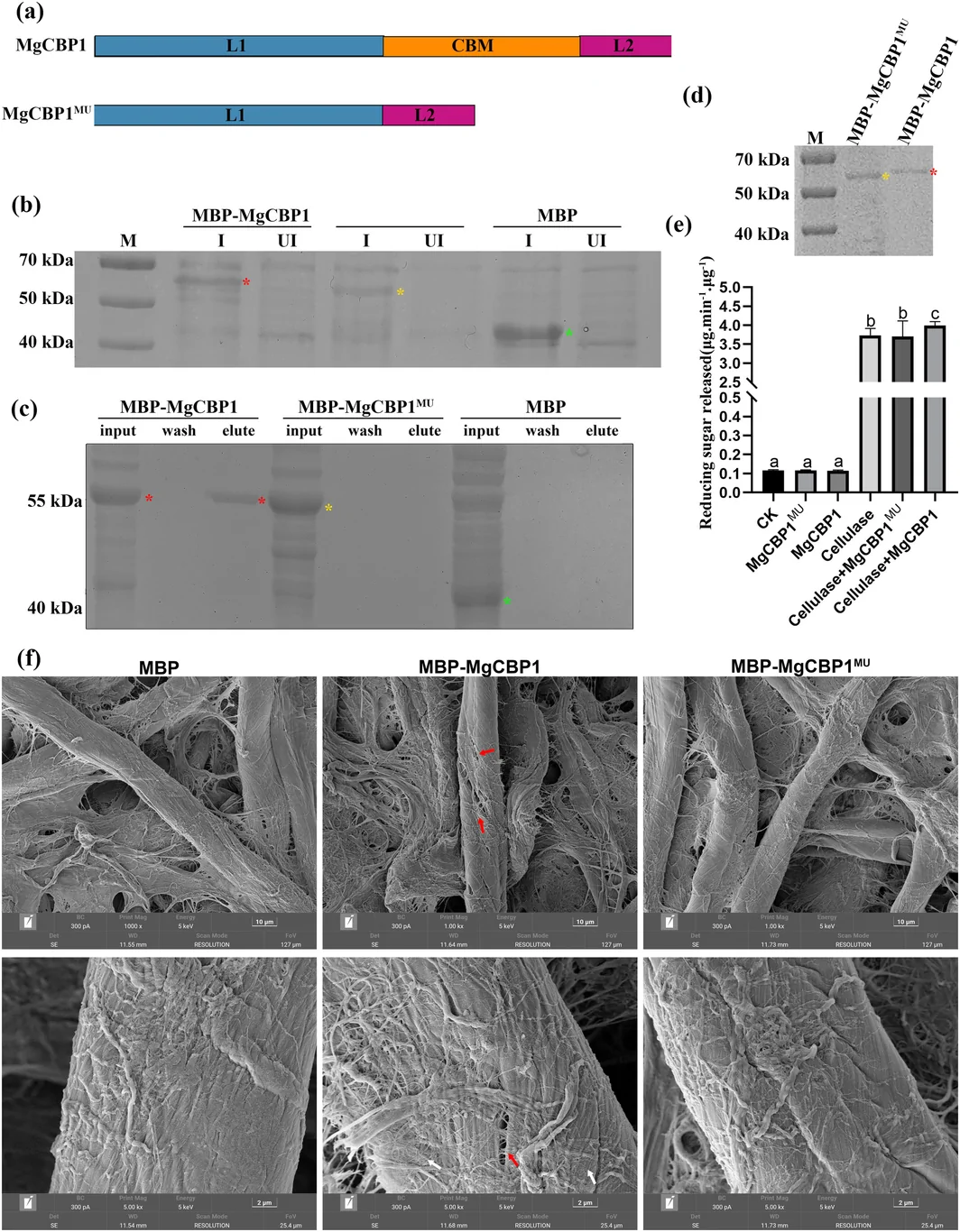
Cellulose‐binding ability and enhancement of cellulolytic efficiency by MgCBP1. (a) Schematic representation of the domain architecture of wild‐type MgCBP1 and the mutant MgCBP1^MU^ lacking the cellulose‐binding module (CBM). L1 and L2 denote linker regions 1 and 2, respectively. (b) SDS‐PAGE analysis of MBP‐MgCBP1, MBP‐MgCBP1^MU^ and MBP expression. UI, uninduced cell lysate; I, induced cell lysate. (c) Cellulose‐binding assay of recombinant MBP‐tagged MgCBP1, MgCBP1^MU^ and MBP analysed by SDS‐PAGE. Input, lysate of *Escherichia coli* cells expressing the recombinant proteins used for incubation with cotton fibres; wash, fraction collected after washing; elute, elution fraction from cotton fibres. (d) SDS‐PAGE of purified recombinant MBP‐tagged MgCBP1 and MgCBP1^MU^. (e) Synergistic effect of MgCBP1 on cellulose hydrolysis by cellulase. Filter papers were incubated with blank control (CK), MgCBP1 alone, MgCBP1^MU^ alone, cellulase alone, cellulase with MgCBP1 and cellulase with MgCBP1^MU^. Hydrolytic activity is expressed as micrograms of reducing sugar released per minute per microgram of protein (μg·min^−1^·μg^−1^). (f) Scanning electron micrographs of filter paper strips incubated with MBP, MBP‐MgCBP1 or MBP‐MgCBP1^MU^. Red arrows point to ruptures and defects on the fibre, while white arrows mark disordered regions of fibril. M, protein molecular weight marker; Asterisks indicate recombinant protein MBP‐MgCBP1 (red), MBP‐MgCBP1^MU^ (yellow) and MBP (green). Data are presented as mean ± standard error from three biological replicates. Different lowercase letters above the bars indicate statistically significant differences (*p* < 0.05).

Sequence analysis revealed that MgCBP1 contains a CBM. To determine whether MgCBP1 actually binds cellulose, an in vitro binding assay was performed using recombinant maltose‐binding protein (MBP)‐tagged MgCBP1, a CBM‐disrupted mutant (MgCBP1^MU^) and MBP as a negative control (Figure 3a,b). After incubation with cotton fibres, a clear protein band corresponding to MBP‐MgCBP1 was detected, whereas no such band was observed for MBP‐MgCBP1^MU^ or the MBP control (Figure 3c).

Recombinant MBP‐tagged MgCBP1 and MgCBP1^MU^ were then purified (Figure 3d) and tested for their effects on cellulose hydrolysis. In filter paper hydrolysis assays, neither protein alone showed detectable hydrolytic activity, and no significant difference from the blank control was observed. However, when cellulase was combined with MgCBP1, the hydrolysis efficiency increased by approximately 16% compared with cellulase alone. By contrast, co‐incubation with cellulase and MgCBP1^MU^ did not significantly improve hydrolysis relative to cellulase alone (Figure 3e).

To further investigate whether MgCBP1 can disrupt cellulose structure, scanning electron microscopy experiments were conducted. After 12 h of incubation with MBP‐tagged MgCBP1, more ruptures and structural defects were observed on the fibre surface. Moreover, compared with the MgCBP1^MU^ and MBP treatments, the MgCBP1‐treated samples exhibited a greater extent of disordered regions with a ‘cotton‐like’ appearance (Figure 3f). Taken together, these results demonstrate that MgCBP1 lacks intrinsic cellulolytic activity but, depending on its intact CBM, binds to cellulose, disrupts the fibril surface integrity, and thereby enhances cellulase‐mediated hydrolysis.

### 2.5 Sequence Analysis of *MgENG1* and *MgENG2*

Two β‐1,4‐endoglucanase genes, designated *MgENG1* and *MgENG2*, were amplified based on the genome sequence of *M. graminicola* . *MgENG1* contains a 969‐bp ORF interrupted by five introns of 275, 12, 129, 323 and 44 bp, whereas *MgENG2* contains a 972‐bp ORF interrupted by four introns of 324, 236, 282 and 49 bp. The ORFs are predicted to encode proteins of 323 and 324 aa, respectively, with calculated molecular masses of 35 kDa each. Both proteins are predicted to possess an N‐terminal signal peptide of 18 aa and to lack a transmembrane domain (Figure S1).

**FIGURE 4:**
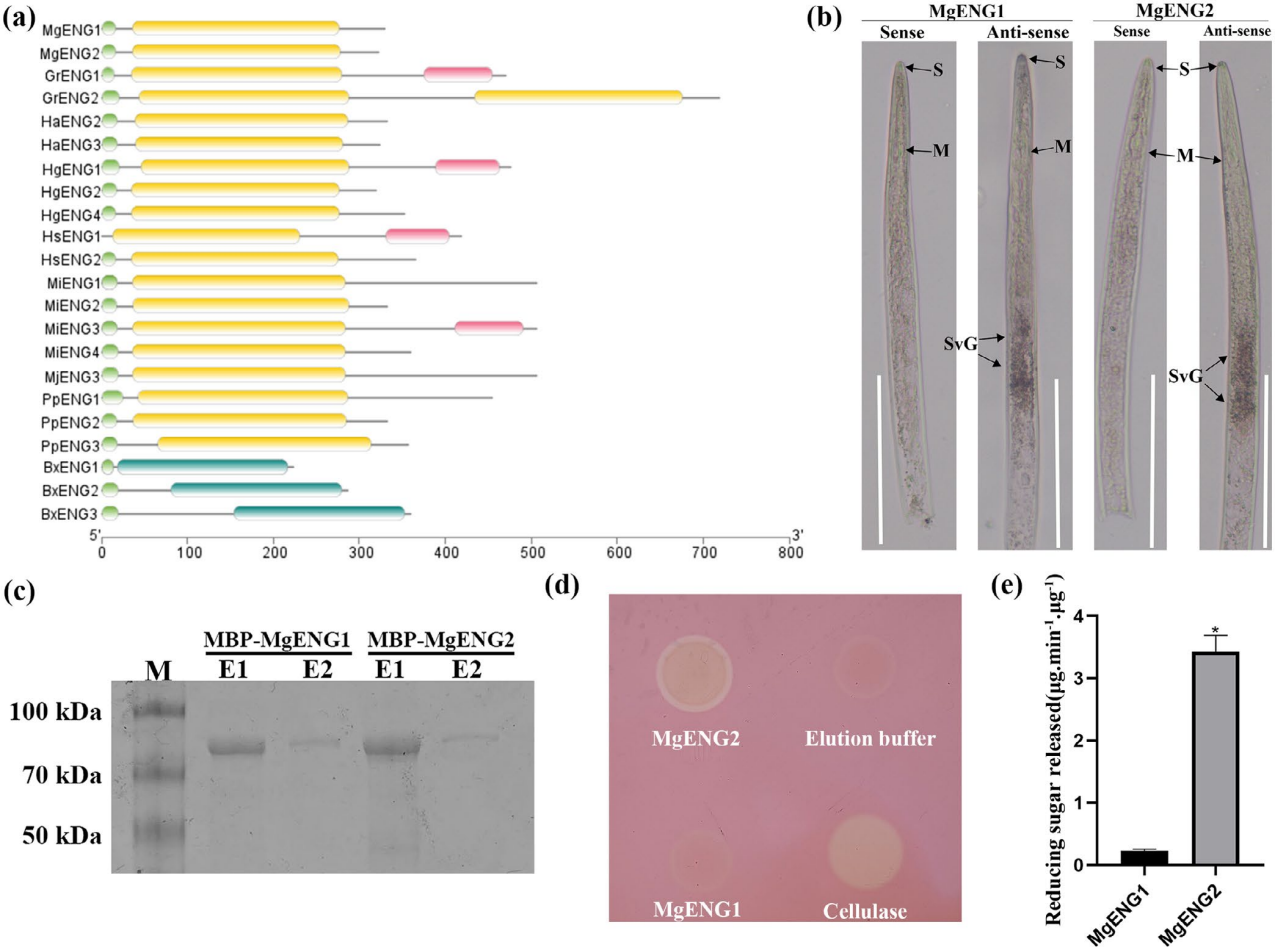
Expression profiles and cellulase activities of MgENG1 and MgENG2. (a) Schematic domain organization of endoglucanase from representative plant‐parasitic nematode species, with green, yellow, pink and cyan representing the signal peptide, glycosyl hydrolase family 5 domain, cellulose‐binding domain and glycosyl hydrolase family 45 domain, respectively. Sequences included are from *Meloidogyne incognita* (MiENG1, AF323087; MiENG2, AAK21881; MiENG3, AY422836; MiENG4, AY422837), *M. javanica* (MjENG3, CAJ77137), *Heterodera glycines* (HgENG1, AF006052; HgENG2, AF006053; HgENG3, AF056048; HgENG4, AY325809), *H. avenae* (HaENG2, JN861114; HaENG3, JN861116), *H. schachtii* (HsENG1, AJ299386; HsENG2, AJ299387), *Globodera rostochiensis* (GrENG1, AF004523; GrENG2, AF004716), *Pratylenchus penetrans* (PpENG1, BAB68522; PpENG2, XCN70975; PpENG3, PV575806) and *Bursaphelenchus xylophilus* (BxENG1, BAD34543; BxENG2, BAD34544; BxENG3, BAD34545). The scale bar indicates amino acid residue positions. (b) In situ localization of *MgENG1* and *MgENG2* in the subventral oesophageal gland cells of pre‐parasitic second‐stage juveniles (pre‐J2s). S, stylet; M, median bulb; SvG, subventral oesophageal gland cells. Scale bar = 100 μm. (c) SDS‐PAGE analysis of purified recombinant MBP‐tagged MgENG1 and MgENG2. E1 and E2, first and second elution fractions from Ni^2+^‐NTA agarose purification, respectively. (d) Qualitative assessment of cellulase activity using a carboxymethyl cellulose plate assay. Cellulase was used as a positive control, and elution buffer served as the negative control. (e) Quantitative measurement of cellulase activity of recombinant MgENG1 and MgENG2 using the 3,5‐dinitrosalicylic acid method. Enzymatic activity is expressed as micrograms of reducing sugar released per minute per microgram of protein (μg·min^−1^·μg^−1^). Data are presented as mean ± standard error from three independent biological replicates. Asterisks indicate a statistically significant difference (*p* < 0.05).

Structural prediction revealed that both MgENG1 and MgENG2 contain a glycosyl hydrolase family 5 (GH5) cellulase domain (residues 34–279). Notably, neither protein possesses a CBM or a linker region, a feature common to most nematode ENGs, although a few orthologs in certain species do contain these domains (Figure 4a). Among *Meloidogyne* orthologs, MgENG1 and MgENG2 showed the highest identity with MiENG4 (AY422837) from *M. incognita* (71.4% and 70.8%, respectively), whereas their identities with MiENG1 (AF323087), MiENG3 (AY422836) from *M. incognita* , and MjENG3 (CAJ77137) from *M. javanica* ranged from 48.9% to 50.2%. When compared with ENGs from other PPNs, including *Heterodera*, *Globodera* and *Pratylenchus* species, the identities ranged from 39.8% to 69.2%, whereas those with BxENG1 (BAD34543) from *Bursaphelenchus xylophilus* were markedly lower (23.1% for MgENG1 and 10.1% for MgENG2) (Figure S1).

### 2.6 MgENG1 and MgENG2 Are Functional Endoglucanases With Distinct Activities

The tissue localization of *MgENG1* and *MgENG2* was examined by in situ hybridization using DIG‐labelled antisense probes in pre‐J2s of *M. graminicola* . Strong hybridization signals were specifically detected in the subventral oesophageal glands, whereas no signal was observed with the corresponding sense probes (Figure 4b).

Recombinant MBP‐tagged proteins were subsequently expressed in *Escherichia coli* and purified (Figure S2; Figure 4c). Cellulase activity was evaluated using both the 3,5‐dinitrosalicylic acid (DNS) method and a carboxymethyl cellulose (CMC) agar plate degradation assay. In the Congo Red‐stained CMC plate assay, MgENG2 produced a clear clearing zone, whereas MgENG1 generated only a faint halo (Figure 4d). Consistently, the DNS assay revealed that MgENG2 exhibited significantly higher enzymatic activity than MgENG1 (Figure 4e). Collectively, these results demonstrate that both MgENG1 and MgENG2 possess endoglucanase activity, with MgENG2 showing substantially stronger cellulose‐degrading capacity.

### 2.7 MgCBP1 Enhances Cellulose Hydrolysis by MgENG2 Through Its CBM

**FIGURE 5:**
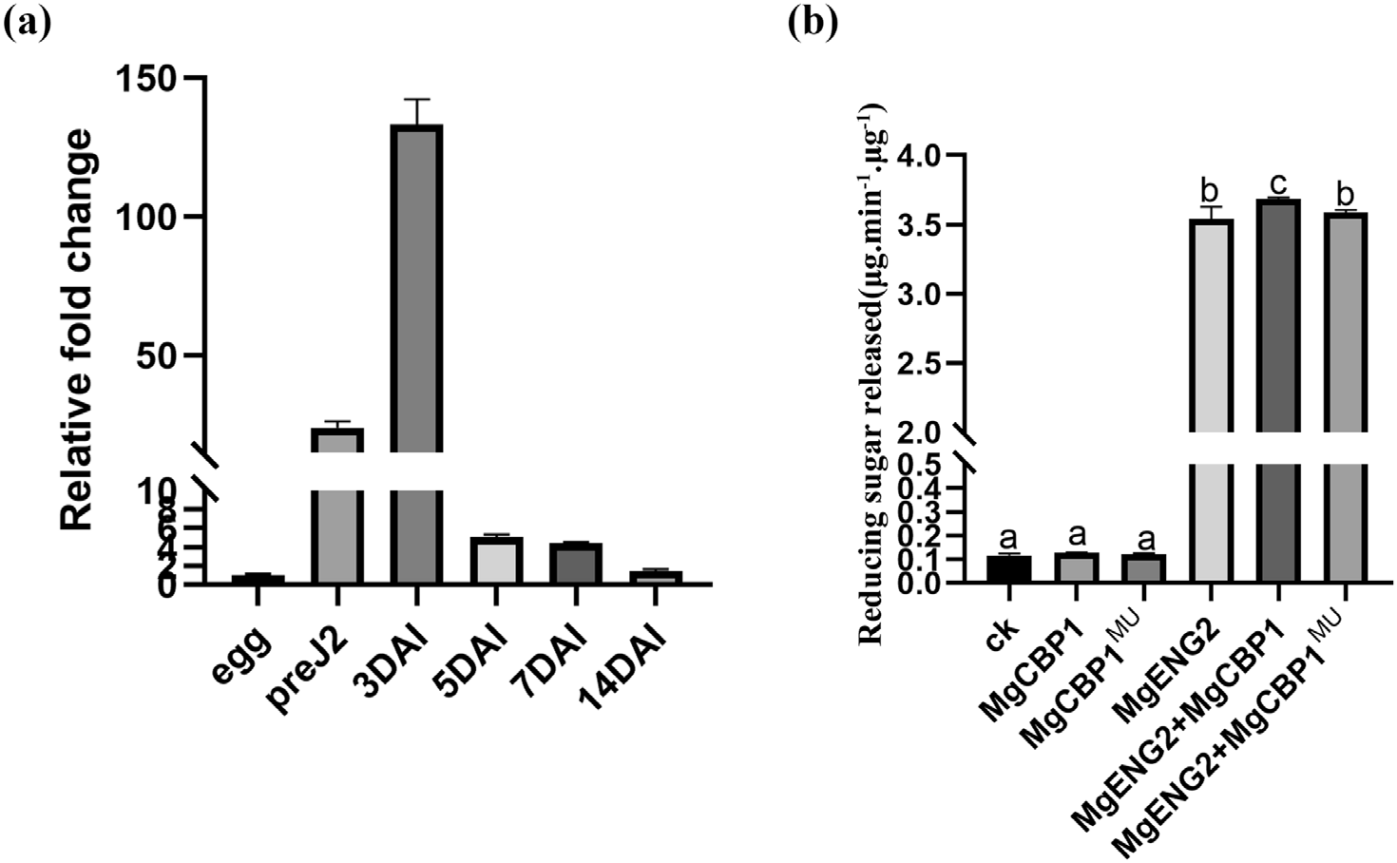
Developmental expression profile of *MgENG2* and enhancement of its cellulolytic efficiency by MgCBP1. (a) Developmental expression profile of *MgENG2* across eggs, pre‐parasitic second‐stage juveniles (pre‐J2s) and four post‐infection time points of *Meloidogyne graminicola*, as determined by reverse transcription‐quantitative PCR. Relative fold change values were calculated using the 2^−ΔΔ*Ct*^ method and normalized to the expression level in eggs. Data are presented as mean ± standard error of three independent biological replicates. DAI, days after inoculation. (b) Synergistic effect of MgCBP1 on cellulose hydrolysis by MgENG2. Filter paper was incubated with blank control (CK), MgCBP1 alone, MgCBP1 lacking the cellulose‐binding module (MgCBP1^MU^) alone, MgENG2 alone, MgENG2 plus MgCBP1 and MgENG2 plus MgCBP1^MU^. Enzymatic activity is expressed as micrograms of reducing sugar released per minute per microgram of protein (μg·min^−1^·μg^−1^). Different lowercase letters above the bars indicate statistically significant differences (*p* < 0.05).

The developmental expression profile of *MgENG2* was further determined by RT‐qPCR. *MgENG2* transcript levels were upregulated in pre‐J2s, with abundance approximately 26‐fold higher than in eggs, and reached a peak at 3 dpi, showing an approximately 148‐fold increase relative to eggs. Thereafter, transcript levels declined sharply to approximately 5‐ and 4‐fold at 5 and 7 dpi, respectively, and returned to egg‐stage levels at 14 dpi (Figure 5a). These findings suggest that MgENG2, like MgCBP1, may function as an effector involved in the invasion and early parasitic stages of *M. graminicola* .

As shown above, MgCBP1 binds cellulose and enhances cellulase‐mediated hydrolysis via its CBM, whereas MgENG2 exhibits strong cellulolytic activity. Given their roles as effectors during early parasitism, we hypothesized that MgENG2 may use MgCBP1 to improve its hydrolysis efficiency. To test this, filter paper hydrolysis assays were performed with various combinations of MgCBP1, MgCBP1^MU^ and MgENG2. The hydrolysis efficiency was significantly higher when MgCBP1 was co‐incubated with MgENG2 than when MgENG2 was incubated alone or with the CBM mutant (Figure 5b), confirming that MgCBP1 promotes MgENG2‐mediated cellulose degradation via its CBM. However, yeast co‐transformation assays showed no physical interaction between MgCBP1 and MgENG2 (Figure S3).

### 2.8 MgCBP1 and MgENG2 Act Synergistically to Promote Nematode Parasitism

**FIGURE 6:**
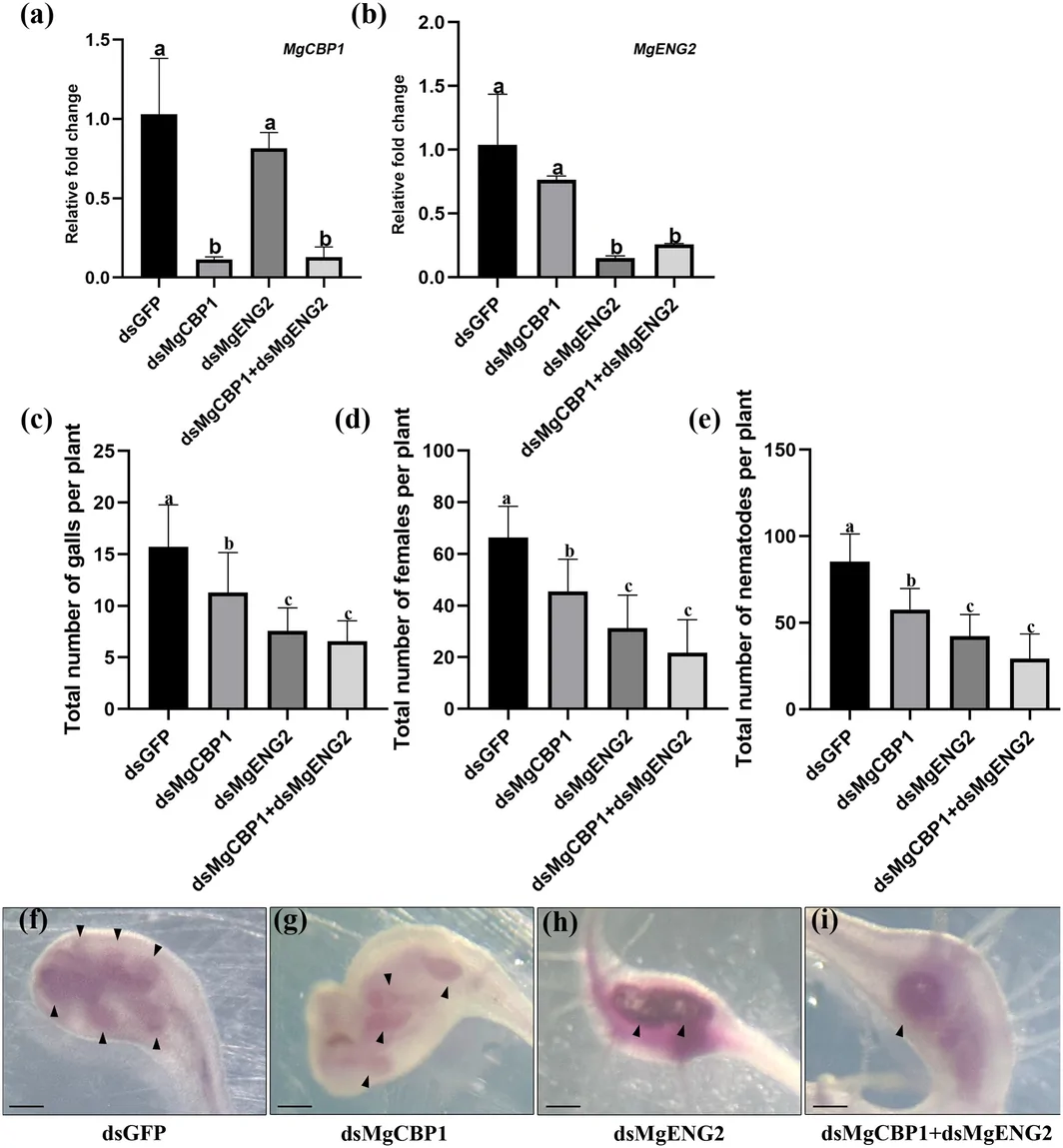
Silencing of *MgCBP1* and *MgENG2* in *Meloidogyne graminicola* attenuates parasitism. (a, b) Relative fold change in *MgCBP1* (a) and *MgENG2* (b) transcript levels in nematodes treated with dsRNA, as determined by reverse transcription‐quantitative PCR, to confirm gene silencing efficiency. dsGFP was used as a control. (c–e) Infection assays showing the numbers of galls (c), females (d) and total nematodes (e) per plant in rice roots inoculated with dsRNA‐treated nematodes. Data are presented as the means ± standard error. Different letters above the bars indicate statistically significant differences (*p* < 0.05). (f–i) Representative images of galls on rice roots at 14 days post‐inoculation with *M. graminicola* after treatment with the indicated dsRNAs. Black arrows indicate nematodes. Scale bars = 200 μm.

To further investigate the functional relevance of MgCBP1‐mediated enhancement of the cellulolytic efficiency of MgENG2 during nematode parasitism, individual and co‐silencing of *MgCBP1* and *MgENG2* were performed via in vitro RNA interference (RNAi). RT‐qPCR analysis confirmed that soaking with *MgCBP1* double‐stranded RNA (dsRNA) significantly reduced *MgCBP1* transcript levels compared to the control (*GFP* dsRNA‐treated group), without significantly affecting *MgENG2* expression. Similarly, *MgENG2* dsRNA treatment reduced its transcript levels, with no significant effect on *MgCBP1* expression. In the co‐silencing treatment, transcript levels of both genes were effectively knocked down (Figure 6a,b). Moreover, none of the treatments affected nematode viability or motility.

Infection assays with dsRNA‐treated nematodes (*dsMgCBP1*, *dsMgENG2* or co‐silencing) revealed significantly reduced numbers of root galls, females and total nematodes in rice roots compared with the *GFP* dsRNA‐treated control. Root gall numbers were decreased by approximately 28%, 51% and 58% in the *dsMgCBP1*, *dsMgENG2* and co‐silencing groups, respectively. Similarly, female numbers per plant were reduced by approximately 31%, 52% and 67%, and total nematode numbers per plant were decreased by approximately 32%, 50% and 66% across the three treatments, respectively. Notably, both co‐silencing and *dsMgENG2* treatments resulted in significantly lower values than the control and *dsMgCBP1* groups; the co‐silencing group showed slightly lower values than the *dsMgENG2* group, although the difference was not statistically significant (Figure 6c–e). Together with our finding that MgCBP1 enhances MgENG2's cellulolytic efficiency via its CBM, these results further support a synergistic relationship between MgENG2 and MgCBP1.

## 3 Discussion

In this study, a CBP gene (*MgCBP1*) and two β‐1,4‐endoglucanase genes (*MgENG1* and *MgENG2*) were cloned from *M. graminicola* . Bioinformatic analysis revealed that MgCBP1 contains a 64‐aa CBM conserved among RKN CBPs, while both MgENG1 and MgENG2 harbour a 245‐aa GH5 glycosyl hydrolase domain conserved among endoglucanases from both RKNs and CNs. All three proteins possess an N‐terminal signal peptide. In situ hybridization further demonstrated that *MgCBP1*, *MgENG1* and *MgENG2* are localized in the subventral oesophageal glands. Signal peptides are typical features of proteins secreted via the endoplasmic reticulum‐Golgi pathway (Elling et al. 2007), and the subventral oesophageal glands are generally recognized as the secretion site for effectors involved in early infection (Rusinque et al. 2021; Haegeman et al. 2012). In agreement with this, RT‐qPCR analysis showed that *MgCBP1* and *MgENG2* were upregulated in pre‐J2s and during the early parasitic stages. Collectively, these findings suggest that MgCBP1 and MgENG2 may function as effectors deployed by *M. graminicola* during invasion and early parasitism.

The plant cell wall serves as the primary physical barrier against pathogen invasion, with cellulose being its major structural component. Disruption of this cellulose framework is therefore often a critical step for successful infection. Previous studies have shown that CBPs can affect the cell wall by binding to cellulose. For example, exogenous application of a low concentration of the CBP from *Clostridium cellulovorans* was found to compete for xyloglucan‐binding sites through cellulose association, thereby loosening the cell wall (Shpigel et al. 1998). Although CBMs have been demonstrated to induce cellulose amorphization and increase substrate accessibility to cellulases (Boraston et al. 2004), no CBP from plant pathogens has yet been reported to enhance cellulase‐mediated cellulose degradation. In this study, recombinant MgCBP1 and a CBM‐disrupted mutant (MgCBP1^MU^) were expressed in *E. coli* and used in cellulose‐binding and hydrolysis assays. The results demonstrated that MgCBP1 possesses cellulose‐binding activity and promotes cellulase‐mediated cellulose degradation, whereas MgCBP1^MU^ lacks this activity, indicating that an intact CBM is required for this enhancing effect. Furthermore, transgenic rice overexpressing *MgCBP1* exhibited enhanced susceptibility to *M. graminicola* compared with wild‐type plants, confirming that MgCBP1 contributes positively to nematode parasitism.

β‐1,4‐endoglucanases are core cellulolytic enzymes secreted by nematodes. Based on phylogenetic and intron position analyses, Ledger et al. (2006) proposed that nematode β‐1,4‐endoglucanases were acquired from bacteria via horizontal gene transfer, and that some of these genes have lost the linker peptide and cellulose‐binding domain during evolution, retaining only the catalytic domain. MgENG1 and MgENG2 from *M. graminicola* similarly contain only a single, highly conserved glycosyl hydrolase catalytic domain. Filter paper hydrolysis and CMC plate assays confirmed that MgENG2 exhibits strong cellulase activity. However, the absence of a CBM has been shown to impair the anchoring of cellulases to insoluble substrates and reduce their hydrolytic efficiency (Hervé et al. 2010). Thus, given that some nematode β‐1,4‐endoglucanases have lost the binding domain during evolution, they may require alternative strategies to compensate for this deficiency. The secretion of independent CBPs, such as MgCBP1 identified in this study, could represent one such key compensatory mechanism.

Few studies have reported synergistic effects between cell wall‐modifying proteins and CWDEs, and most of these reports focus on expansins and cellulases. For example, the tomato expansin SlEXP1 cooperates with the endoglucanase SlCel2 to target the xylan‐cellulose network, thereby promoting cellulose degradation and wall loosening during fruit softening (Su et al. 2024; Li et al. 2025). Similarly, the expansin BsEXLX1 from *B. subtilis* enhances cellulase‐mediated cellulose degradation (Kim et al. 2009). However, direct experimental evidence for synergy between a cellulase and a CBP in plant pathogens has not yet been reported. In this study, filter paper hydrolysis assays demonstrated that co‐incubation of MgCBP1 with MgENG2 significantly increased cellulose degradation compared with MgENG2 alone, and this enhancement required an intact CBM in MgCBP1. Scanning electron microscopy revealed that MgCBP1 caused structural disruption and increased disordered regions on the fibre surface in a CBM‐dependent manner. Yeast co‐transformation assays did not detect any physical interaction between MgCBP1 and MgENG2. RT‐qPCR analysis showed that both genes were upregulated in pre‐parasitic and early parasitic stages of *M. graminicola* , exhibiting distinct sequential patterns, with *MgCBP1* expression higher in the pre‐parasitic stage and *MgENG2* higher in the early parasitic stage. CBMs have been proposed to facilitate cellulase‐mediated hydrolysis by both disrupting the ordered cellulose structure and increasing the local enzyme concentration at the substrate surface (Bernardes et al. 2019). Our findings support this mechanistic model, suggesting that MgCBP1 binds and alters the cellulose surface, indirectly increasing the accessibility of MgENG2 to the substrate. The sequential expression pattern of both genes further suggests that MgCBP1 may act as an initial ‘scout’ that disrupts the cellulose architecture at the root surface during invasion, thereby facilitating the subsequent action of MgENG2 during early parasitism.

We further performed RNAi‐mediated silencing assays to assess the in vivo relevance of this synergistic effect. Individual silencing of *MgENG2* and co‐silencing of both genes significantly reduced nematode parasitism, with the co‐silenced group showing lower infectivity than the *MgENG2*‐silenced group, although the difference was not statistically significant. By contrast, silencing of *MgCBP1* alone also significantly impaired parasitism, but to a markedly lesser extent than the other two treatments. These results indicate that MgENG2 serves as a key effector mediating nematode infection, whereas MgCBP1 plays a subordinate role, primarily acting to enhance MgENG2's cellulolytic efficiency.

In summary, we have, for the first time, identified a cellulose‐binding effector protein (MgCBP1) and a β‐1,4‐endoglucanase (MgENG2) that possesses strong cellulolytic activity from *M. graminicola* . Both genes are upregulated in pre‐J2s and during early parasitism. Notably, MgCBP1 enhances the cellulose‐degradation efficiency of MgENG2 through synergistic action, thereby promoting infection and parasitism. To our knowledge, this study provides the first experimental evidence for synergy between a β‐1,4‐endoglucanase and a CBP in plant pathogens. These findings not only offer a mechanistic explanation for the functional compensation of nematode β‐1,4‐endoglucanases that have lost their CBMs during evolution, but also deepen our understanding of PPN infection strategies involving cell wall modification. Furthermore, the demonstrated synergy between MgCBP1 and MgENG2 may provide promising targets for nematode control through multigene silencing.

## 4 Experimental Procedures

### 4.1 Nematode and Plant Materials

The *M. graminicola* population was isolated from cultivated rice in Hainan, China, established from a single egg mass, and maintained on rice ( *O. sativa* ‘Nipponbare’) roots at 28°C. Pre‐J2s were obtained by hatching eggs as described previously (Huang et al. 2010).

### 4.2 Genes Cloning and Sequences Analysis

Total RNA was extracted from approximately 20,000 pre‐J2s using the RNAprep Pure Micro Kit (TianGen Biotech). First‐strand cDNA was synthesized with the TransScript One‐Step gDNA Removal and cDNA Synthesis Super Mix (TransGen Biotech) following the manufacturer's protocol. The full‐length cDNA sequences of *MgCBP1*, *MgENG1* and *MgENG2* were amplified using the specific primer pairs MgCBP1‐F/R, MgENG1‐F/R and MgENG2‐F/R, respectively, which were designed based on the corresponding gene sequences. All primers used in this study are listed in Table S1.

For sequence analysis, the deduced protein sequences were used as queries to search for homologous sequences in the NCBI BLAST database (https://www.ncbi.nlm.nih.gov/BLAST/) and the WormBase ParaSite database (https://parasite.wormbase.org/). Signal peptides and transmembrane domains were predicted using SignalP v. 4.1 (https://www.cbs.dtu.dk/services/SignalP/) and TMHMM v. 2.0 (https://www.cbs.dtu.dk/services/TMHMM/), respectively. Multiple sequence alignment was performed with MAFFT and visualized with Jalview (Waterhouse et al. 2009), and sequence similarities among the aligned sequences were subsequently calculated using TBtools. Conserved domains were predicted with InterPro (https://www.ebi.ac.uk/interpro/) and visualized with TBtools (Chen et al. 2023).

### 4.3 In Situ Hybridization

Sense and antisense probes were synthesized as described previously (Vanholme et al. 2002). A 187‐bp fragment of *MgCBP1*, a 204‐bp fragment of *MgENG1* and a 229‐bp fragment of *MgENG2* were selected from the full‐length sequences as target regions. Templates for sense and antisense probes were generated by PCR using the primer pairs MgCBP1‐ISH‐F/R, MgENG1‐ISH‐F/R and MgENG2‐ISH‐F/R. Probe labelling was performed according to the manufacturer's protocol of the DIG RNA Labelling Mix (Roche). For in situ hybridization, approximately 10,000 freshly hatched pre‐J2s were used, and the assay was conducted following the method of Jaouannet et al. (2012). The results were observed and photographed using an Eclipse 90i microscope (Nikon).

### 4.4 Developmental Expression Analysis

Pre‐J2s of *M. graminicola* were inoculated into rice roots. To synchronize the infection process, the inoculated plants were transplanted into fresh soil 24 h after inoculation. Total RNA was extracted from approximately 100 nematodes at each indicated stage (eggs, pre‐J2s, and four post‐infection time points) using the RNAprep Pure Micro Kit (TianGen Biotech). First‐strand cDNA was synthesized with the TransScript All‐in‐One First‐Strand cDNA Synthesis SuperMix (TransGen Biotech). qPCR was performed as previously described (Haegeman et al. 2013), with *MgActin2* used as the internal reference gene (Chen et al. 2017).

### 4.5 Construction of the CBM‐Deleted MgCBP1 Mutant

A mutant form of MgCBP1 lacking the CBM domain (designated MgCBP1^MU^) was generated via overlap extension PCR (Horton et al. 1989). Briefly, specific overlapping primers, CBP1‐CBMwo‐F and CBP1‐CBMwo‐R, were designed flanking the CBM‐coding region. In the first round of PCR, two truncated fragments of MgCBP1 without the CBM domain were separately amplified using the primer pairs CBP1‐F/CBP1‐CBMwo‐R and CBP1‐CBMwo‐F/CBP1‐R, respectively. In the second round of PCR, the two first‐round products were used as templates and assembled into a single fragment with the primer pair CBP1‐F/CBP1‐R, yielding the CBM‐deleted MgCBP1 construct (MgCBP1^MU^).

### 4.6 Expression and Purification of Recombinant Proteins

The sequences of *MgCBP1*, *MgCBP1* ^*MU*^, *MgENG1* and *MgENG2* were cloned into the pET28a vector with an N‐terminal MBP tag to generate recombinant protein expression constructs. These recombinant proteins were produced in *E. coli* Rosetta (DE3) cells and purified using Ni^2+^‐NTA agarose (TransGen Biotech) according to the manufacturer's protocol. The concentration and purity of the purified proteins were determined by the BCA assay (Tiangen Biotech) and SDS‐PAGE, respectively.

### 4.7 Cellulose Binding Assay

The cellulose‐binding assay was performed according to Lehtiö et al. (2001) with minor modifications. Briefly, 200 μg of recombinant protein was incubated with 1 mg of cotton fibres in 1 mL of phosphate‐buffered saline (PBS, pH 7.4) at 37°C for 1.5 h. After incubation, the fibres were washed three times with PBS to remove unbound proteins. Bound proteins were then eluted by heating in 10% (w/v) SDS at 130°C for 10 min, and the eluates were analysed by SDS‐PAGE. Recombinant MgCBP1^MU^ and MBP were included as negative controls.

### 4.8 Cellulase Activity Assays

Cellulase activity was evaluated using two methods: a CMC agar plate assay and a filter paper hydrolysis assay. The CMC agar plate assay was performed as described by Teather and Wood (1982) with minor modifications. Briefly, CMC agarose plates were prepared by supplementing basal medium with 0.1% (w/v) CMC and 0.8% (w/v) agarose. Recombinant protein or commercial cellulase C0057 (TCI Chemicals) was spotted onto the plates and incubated at 37°C for 30 min. After incubation, the plates were stained with 0.1% (w/v) Congo Red solution at room temperature for 10–15 min. The dye was then removed, and the plates were rinsed with 1 M NaCl for 1–2 min to decolourize until clear hydrolysis zones became visible.

The filter paper hydrolysis assay was performed according to Ghose (1987) with modification. Briefly, filter paper strips (1 × 1 cm) were incubated with recombinant protein or commercial cellulase C0057 in 500 μL of 3 M sodium citrate buffer (pH 5.2) at 40°C for 2 h. Subsequently, 1 mL of 3,5‐dinitrosalicylic acid (DNS) reagent was added to terminate the reaction, and the mixture was boiled at 120°C for 5 min. After cooling to room temperature, the absorbance of the reducing sugars was measured at 540 nm.

### 4.9 Generation of Transgenic Rice Plants

Transgenic rice lines were generated as described previously (Huang et al. 2023). The coding sequence of *MgCBP1* was cloned into the pUbi vector as described by Chen et al. (2017). The overexpression constructs were introduced into *Agrobacterium tumefaciens* EHA105 through the CaCl_2_‐mediated transformation method, and the resulting strain was used to infect callus tissues induced from seeds of the *japonica* rice cultivar Nipponbare. Transformants were selected on N6 medium supplemented with 50 mg/L hygromycin.

The expression of *MgCBP1* in each transgenic rice line was determined by RT‐qPCR. Total RNA was extracted from rice roots using the RNAprep Pure Micro Kit (TianGen Biotech), and first‐strand cDNA was synthesized with the TransScript All‐in‐One First‐Strand cDNA Synthesis SuperMix (TransGen Biotech) according to the manufacturer's protocol.

### 4.10 In Vitro RNAi

dsRNAs targeting *MgCBP1*, *MgENG2* and *GFP* were synthesized in vitro using the MEGAscript RNAi Kit (Thermo Fisher Scientific) according to the manufacturer's protocol. For *MgCBP1*, the sense and antisense directions of a 334‐bp fragment appending the T7 sequence in the 5′ terminus were generated by two separate PCR amplifications using the primer pairs T7‐CBP1‐RNAi‐F1/R2 and T7‐CBP1‐RNAi‐F2/R1. Similarly, a 229‐bp *MgENG2* fragment and a 447‐bp GFP fragment appending the T7 sequence in the 5′ terminus were amplified using the primer pairs T7‐ENG2‐RNAi‐F/ENG2‐RNAi‐R, ENG2‐RNAi‐F/T7‐ENG2‐RNAi‐R, T7‐GFP‐F/GFP‐R and GFP‐F/T7‐GFP‐R. The dsRNAs for *MgCBP1*, *MgENG2* and *GFP* were then synthesized following the MEGAscript RNAi Kit protocol.

Soaking‐mediated RNAi was performed as described previously (Bakhetia et al. 2005; Rosso et al. 2005) with minor modifications. Briefly, freshly hatched J2s were soaked in a solution containing 2 mg/mL dsRNA at 25°C for 4 h. After treatment, the nematodes were washed with nuclease‐free water to remove externally adsorbed dsRNA. To evaluate gene silencing efficiency, total RNA was extracted from the treated J2s, and the transcript levels of *MgCBP1* and *MgENG2* were determined by RT‐qPCR as described above.

### 4.11 Infection Assay

The infection assay was performed as described by Chen et al. (2017) with minor modifications. Briefly, 14‐day‐old rice plants were each inoculated with 200 pre‐J2s of *M. graminicola* . At 24 hpi, the plants were transferred to fresh sand‐soil mixture to synchronize the infection process. Roots were harvested at 14 dpi, stained with acid fuchsin and preserved in acidified glycerol. The numbers of females and juveniles inside the roots were counted for each plant.

### 4.12 Scanning Electron Microscopy

Filter paper strips (1 × 1 cm) were incubated separately with recombinant protein MBP, MgCBP1 or MgCBP1^MU^ at 2 μg/mL for 12 h at 37°C. The strips were then collected, dried at 37°C for 24 h and coated with platinum (approximately 10 nm thick) on a SC7620 sputter coater (Quorum Technologies) at 10 mA per 45 s. Images were captured using a scanning electron microscope (Tescan).

### 4.13 Yeast Co‐Transformation Assay

The sequence of *MgCBP1* ^ΔSP^ was cloned into the pGBKT7 vector as bait and the sequence of *MgENG2* ^ΔSP^ was cloned into the pGADT7 vector as the prey. The resulting constructs were co‐transformed into *Saccharomyces cerevisiae* Y2HGold following the manufacturer's protocol (Clontech). The interaction between MgCBP1 and MgENG2 was determined by yeast growth on SD/−Trp/−Leu/−His/−Ade selection medium.

### 4.14 Statistical Analysis

All statistical analyses were performed using GraphPad Prism v. 8.0.2 (GraphPad Software). One‐way ANOVA with Dunnett's multiple comparisons test was used for comparisons of multiple treatment groups against a control group, and a two‐tailed Student's *t*‐test was applied for pairwise comparisons.

## Author Contributions

**Chunhui Huang:** methodology, investigation, writing – original draft. **Yuqing Cao:** methodology, investigation. **Ao Xu:** formal analysis, software. **Jiani Chen:** investigation. **Borong Lin:** methodology, funding acquisition. **Qiuling Huang:** investigation, formal analysis, funding acquisition. **Kan Zhuo:** conceptualization, funding acquisition, resources, writing – review and editing.

## Funding

This work was supported by grants from Department of Science and Technology of Guangdong Province (2026B0202190006), the National Natural Science Foundation of China (32272490, 32502421 and 32572769), the Earmarked Fund for Modern Agro‐industry Technology Research System (nycytx‐001) and the Shaanxi Innovation Team Project (2024RS‐CXTD‐73).

## Conflicts of Interest

The authors declare no conflicts of interest.

## Supporting information


**Figure S1:** Multiple sequence alignment of endo‐β‐1,4‐glucanase proteins from plant‐parasitic nematodes. The amino acid sequences were aligned and shaded in blue according to conservation level, with darker shades indicating higher identity. The green dashed box marks the predicted N‐terminal signal peptide, while the red dashed boxes delineate the conserved glycoside hydrolase family 5 catalytic domain. Sequences included are from *Meloidogyne incognita* (MiENG1, AF323087; MiENG2, AAK21881; MiENG3, AY422836; MiENG4, AY422837), *M. javanica* (MjENG3, CAJ77137), *Heterodera glycines* (HgENG1, AF006052; HgENG2, AF006053; HgENG3, AF056048; HgENG4, AY325809), *H. avenae* (HaENG2, JN861114; HaENG3, JN861116), *H. schachtii* (HsENG1, AJ299386; HsENG2, AJ299387), *Globodera rostochiensis* (GrENG1, AF004523; GrENG2, AF004716), *Pratylenchus penetrans* (PpENG1, BAB68522; PpENG2, XCN70975; PpENG3, PV575806) and *Bursaphelenchus xylophilus* (BxENG1, BAD34543; BxENG2, BAD34544; BxENG3, BAD34545).

**Figure S2:** SDS‐PAGE analysis of recombinant MBP‐MgENG1 and MBP‐MgENG2 expression and purification, with M indicating the protein molecular weight marker, UI the uninduced cell lysate, I the induced cell lysate, E1 and E2 the first and second elution fractions, respectively, and with the recombinant proteins MBP‐MgENG1 and MBP‐MgENG2 indicated by red and yellow dashed boxes, respectively.

**Figure S3:** Yeast co‐transformation assay of MgCBP1 and MgENG2. The indicated bait (pGBKT7) and prey (pGADT7) plasmids were co‐transformed into yeast strain Y2HGold. Transformants were selected on SD/−Leu/−Trp medium for 2 days, and protein interaction was assessed by spotting onto SD/−Ade/−His/−Leu/−Trp medium.

**Table S1:** Primers used in this study.

## Data Availability

The data supporting the findings of this study are available in the Supporting Information of this article.

## References

[mpp70346-bib-0001] Bakhetia, M. , W. Charlton , H. J. Atkinson , and M. McPherson . 2005. “RNA Interference of Dual Oxidase in the Plant Nematode *Meloidogyne incognita* .” Molecular Plant–Microbe Interactions 18: 1099–1106. 10.1094/MPMI-18-1099 16255249

[mpp70346-bib-0002] Bernardes, A. , V. Pellegrini , F. Curtolo , et al. 2019. “Carbohydrate Binding Modules Enhance Cellulose Enzymatic Hydrolysis by Increasing Access of Cellulases to the Substrate.” Carbohydrate Polymers 211: 57–68. 10.1016/j.carbpol.2019.01.108 30824104

[mpp70346-bib-0003] Boraston, A. B. , D. N. Bolam , H. J. Gilbert , et al. 2004. “Carbohydrate‐Binding Modules: Fine‐Tuning Polysaccharide Recognition.” Biochemical Journal 382: 769–781. 10.1042/BJ20040892 PMC1133952 15214846

[mpp70346-bib-0004] Brás, J. L. , A. Cartmell , A. L. M. Carvalho , et al. 2011. “Structural Insights Into a Unique Cellulase Fold and Mechanism of Cellulose Hydrolysis.” Proceedings of the National Academy of Sciences of the United States of America 108: 5237–5242. 10.1073/pnas.1015006108 PMC3069175 21393568

[mpp70346-bib-0005] Chen, C. , Y. Wu , J. Li , et al. 2023. “TBtools‐II: A ‘One for All, All for One’ Bioinformatics Platform for Biological Big‐Data Mining.” Molecular Plant 16: 1733–1742. 10.1016/j.molp.2023.09.010 37740491

[mpp70346-bib-0007] Chen, J. , B. Lin , Q. Huang , L. Hu , K. Zhuo , and J. Liao . 2017. “A Novel *Meloidogyne graminicola* Effector, MgGPP, Is Secreted Into Host Cells and Undergoes Glycosylation in Concert With Proteolysis to Suppress Plant Defenses and Promote Parasitism.” PLoS Pathogens 13: e1006301. 10.1371/journal.ppat.1006301 PMC5402989 28403192

[mpp70346-bib-0008] Ding, X. , J. Shields , R. Allen , and R. S. Hussey . 1998. “A Secretory Cellulose‐Binding Protein cDNA Cloned From the Root‐Knot Nematode (*Meloidogyne incognita*).” Molecular Plant–Microbe Interactions 11: 952–959. 10.1094/MPMI.1998.11.10.952 9768512

[mpp70346-bib-0009] Elling, A. A. , E. L. Davis , R. S. Hussey , and T. J. Baum . 2007. “Active Uptake of Cyst Nematode Parasitism Proteins Into the Plant Cell Nucleus.” International Journal for Parasitology 37: 1269–1279. 10.1016/j.ijpara.2007.03.012 17517414

[mpp70346-bib-0010] Eves‐Van Den Akker, S. , B. Stojilković , and G. Gheysen . 2021. “Recent Applications of Biotechnological Approaches to Elucidate the Biology of Plant–Nematode Interactions.” Current Opinion in Biotechnology 70: 122–130. 10.1016/j.copbio.2021.03.008 33932862

[mpp70346-bib-0011] Gao, B. , R. Allen , E. L. Davis , T. J. Baum , and R. S. Hussey . 2004a. “Developmental Expression and Biochemical Properties of a β‐1,4‐Endoglucanase Family in the Soybean Cyst Nematode, *Heterodera glycines* .” Molecular Plant Pathology 5: 93–104. 10.1111/j.1364-3703.2004.00209.x 20565586

[mpp70346-bib-0012] Gao, B. , R. Allen , E. L. Davis , T. J. Baum , and R. S. Hussey . 2004b. “Molecular Characterisation and Developmental Expression of a Cellulose‐Binding Protein Gene in the Soybean Cyst Nematode *Heterodera glycines* .” International Journal for Parasitology 34: 1377–1383. 10.1016/j.ijpara.2004.09.001 15542098

[mpp70346-bib-0013] Ghose, T. K. 1987. “Measurement of Cellulase Activities.” Pure and Applied Chemistry 59: 257–268.

[mpp70346-bib-0014] Haegeman, A. , L. Bauters , T. Kyndt , M. M. Rahman , and G. Gheysen . 2013. “Identification of Candidate Effector Genes in the Transcriptome of the Rice Root Knot Nematode *Meloidogyne graminicola* .” Molecular Plant Pathology 14: 379–390. 10.1111/mpp.12014 PMC6638898 23279209

[mpp70346-bib-0015] Haegeman, A. , S. Mantelin , J. T. Jones , and G. Gheysen . 2012. “Functional Roles of Effectors of Plant‐Parasitic Nematodes.” Gene 492: 19–31. 10.1016/j.gene.2011.10.040 22062000

[mpp70346-bib-0016] Hall, J. , G. W. Black , L. M. A. Ferreira , et al. 1995. “The Non‐Catalytic Cellulose‐Binding Domain of a Novel Cellulase From *Pseudomonas fluorescens* subsp. *cellulosa* Is Important for the Efficient Hydrolysis of Avicel.” Biochemical Journal 309: 749–756. 10.1042/bj3090749 PMC1135696 7639689

[mpp70346-bib-0017] Hervé, C. , A. Rogowski , A. W. Blake , S. E. Marcus , H. J. Gilbert , and J. P. Knox . 2010. “Carbohydrate‐Binding Modules Promote the Enzymatic Deconstruction of Intact Plant Cell Walls by Targeting and Proximity Effects.” Proceedings of the National Academy of Sciences of the United States of America 107: 15293–15298. 10.1073/pnas.1005732107 PMC2930570 20696902

[mpp70346-bib-0018] Hewezi, T. , P. Howe , T. R. Maier , et al. 2008. “Cellulose Binding Protein From the Parasitic Nematode *Heterodera schachtii* Interacts With *Arabidopsis* Pectin Methylesterase: Cooperative Cell Wall Modification During Parasitism.” Plant Cell 20: 3080–3093. 10.1105/tpc.108.063065 PMC2613657 19001564

[mpp70346-bib-0019] Horton, R. M. , H. D. Hunt , S. N. Ho , J. K. Pullen , and L. R. Pease . 1989. “Engineering Hybrid Genes Without the Use of Restriction Enzymes: Gene Splicing by Overlap Extension.” Gene 77: 61–68. 10.1016/0378-1119(89)90359-4 2744488

[mpp70346-bib-0020] Hu, L. , R. Cui , L. Sun , B. Lin , K. Zhuo , and J. Liao . 2013. “Molecular and Biochemical Characterization of the β‐1, 4‐Endoglucanase Gene *Mj‐Eng‐3* in the Root‐Knot Nematode *Meloidogyne javanica* .” Experimental Parasitology 135: 15–23. 10.1016/j.exppara.2013.05.012 23747693

[mpp70346-bib-0021] Huang, G. , R. Dong , R. Allen , et al. 2010. “Two Chorismate Mutase Genes From the Root‐Knot Nematode *Meloidogyne incognita* .” Molecular Plant Pathology 6: 23–30. 10.1111/j.1364-3703.2004.00257.x 20565635

[mpp70346-bib-0022] Huang, Q. , B. Lin , Y. Cao , et al. 2023. “CRISPR/Cas9‐Mediated Mutagenesis of the Susceptibility Gene *OsHPP04* in Rice Confers Enhanced Resistance to Rice Root‐Knot Nematode.” Frontiers in Plant Science 14: 1134653. 10.3389/fpls.2023.1134653 PMC10043372 36998699

[mpp70346-bib-0023] Jaouannet, M. , L. Perfus Barbeoch , E. Deleury , et al. 2012. “A Root‐Knot Nematode Secreted Protein Is Injected Into Giant Cells and Targeted to the Nuclei.” New Phytologist 194: 924–931. 10.1111/j.1469-8137.2012.04164.x 22540860

[mpp70346-bib-0024] Kim, E. S. , H. J. Lee , W. G. Bang , I. G. Choi , and K. H. Kim . 2009. “Functional Characterization of a Bacterial Expansin From *Bacillus subtilis* for Enhanced Enzymatic Hydrolysis of Cellulose.” Biotechnology and Bioengineering 102: 1342–1353. 10.1002/bit.22193 19058186

[mpp70346-bib-0026] Ledger, T. N. , S. Jaubert , N. Bosselut , P. Abad , and M. N. Rosso . 2006. “Characterization of a New β‐1,4‐Endoglucanase Gene From the Root‐Knot Nematode *Meloidogyne incognita* and Evolutionary Scheme for Phytonematode Family 5 Glycosyl Hydrolases.” Gene 382: 121–128. 10.1016/j.gene.2006.06.023 16962258

[mpp70346-bib-0027] Lehtiö, J. , H. Wernérus , P. Samuelson , T. T. Teeri , and S. Ståhl . 2001. “Directed Immobilization of Recombinant Staphylococci on Cotton Fibers by Functional Display of a Fungal Cellulose‐Binding Domain.” FEMS Microbiology Letters 195: 197–204. 10.1111/j.1574-6968.2001.tb10521.x 11179652

[mpp70346-bib-0028] Li, X. , D. Xu , L. Zhang , and L. Zhao . 2025. “Endoglucanase SlCel2 and Expansin SlExp1 Synergistically Affect Cellulose Degrading and Tomato Fruit Softening.” BMC Plant Biology 25: 704. 10.1186/s12870-025-06749-7 PMC12107895 40419969

[mpp70346-bib-0029] Liu, Y. , J. Li , M. Ye , et al. 2026. “ *Hirschmanniella mucronata* Effector HmENG1 Interacts With OsGAPDH to Promote Nematode Infection in Rice.” Phytopathology 116: 1290–1300. 10.1094/PHYTO-12-25-0393. 41842444

[mpp70346-bib-0030] Long, H. , D. Peng , W. Huang , H. Peng , and G. Wang . 2013. “Molecular Characterization and Functional Analysis of Two New β‐1,4‐Endoglucanase Genes (*Ha‐Eng‐2*, *ha‐Eng‐3*) From the Cereal Cyst Nematode *Heterodera avenae* .” Plant Pathology 62: 953–960.

[mpp70346-bib-0031] Mejias, J. , J. Bazin , N. Truong , et al. 2021. “The Root‐Knot Nematode Effector MiEFF18 Interacts With the Plant Core Spliceosomal Protein smd1 Required for Giant Cell Formation.” New Phytologist 229: 3408–3423. 10.1111/nph.17089 33206370

[mpp70346-bib-0032] Rehman, S. , P. Butterbach , H. Popeijus , et al. 2009. “Identification and Characterization of the Most Abundant Cellulases in Stylet Secretions From *Globodera rostochiensis* .” Phytopathology 99: 194–202. 10.1094/PHYTO-99-2-0194 19245333

[mpp70346-bib-0033] Rosso, M. , M. Dubrana , N. Cimbolini , et al. 2005. “Application of RNA Interference to Root‐Knot Nematode Genes Encoding Esophageal Gland Proteins.” Molecular Plant–Microbe Interactions 18: 615–620. 10.1094/MPMI-18-0615 16042006

[mpp70346-bib-0034] Rusinque, L. , C. Maleita , I. Abrantes , J. E. Palomares‐Rius , and M. L. Inácio . 2021. “ *Meloidogyne graminicola*—A Threat to Rice Production: Review Update on Distribution, Biology, Identification, and Management.” Biology 10: 1163. 10.3390/biology10111163 PMC8614973 34827156

[mpp70346-bib-0035] Shibuya, H. , and T. Kikuchi . 2008. “Purification and Characterization of Recombinant Endoglucanases From the Pine Wood Nematode *Bursaphelenchus xylophilus* .” Bioscience Biotechnology and Biochemistry 72: 1325–1332. 10.1271/bbb.70819 18460801

[mpp70346-bib-0036] Shpigel, E. , L. Roiz , R. Goren , and O. Shoseyov . 1998. “Bacterial Cellulose‐Binding Domain Modulates In Vitro Elongation of Different Plant Cells.” Plant Physiology 117: 1185–1194. 10.1104/pp.117.4.1185 PMC34883 9701575

[mpp70346-bib-0037] Smant, G. , J. P. Stokkermans , Y. Yan , et al. 1998. “Endogenous Cellulases in Animals: Isolation of β‐1,4‐Endoglucanase Genes From Two Species of Plant‐Parasitic Cyst Nematodes.” Proceedings of the National Academy of Sciences of the United States of America 95: 4906–4911. 10.1073/pnas.95.9.4906 PMC20186 9560201

[mpp70346-bib-0038] Su, G. , Y. Lin , C. Wang , et al. 2024. “Expansin SlExp1 and Endoglucanase SlCel2 Synergistically Promote Fruit Softening and Cell Wall Disassembly in Tomato.” Plant Cell 36: 709–726. 10.1093/plcell/koad291 PMC10896287 38000892

[mpp70346-bib-0039] Teather, R. M. , and P. J. Wood . 1982. “Use of Congo Red–Polysaccharide Interactions in Enumeration and Characterization of Cellulolytic Bacteria From the Bovine Rumen.” Applied and Environmental Microbiology 43: 777–780. 10.1128/aem.43.4.777-780.1982 PMC241917 7081984

[mpp70346-bib-0040] Uehara, T. , A. Kushida , and Y. Momota . 2001. “PCR‐Based Cloning of Two β‐1,4‐Endoglucanases From the Root‐Lesion Nematode *Pratylenchus penetrans* .” Nematology 3: 335–341.

[mpp70346-bib-0041] Vanholme, B. , J. D. Meutter , T. Tytgat , J. de Meutter , G. Gheysen , and I. Vanhoutte . 2002. “An Improved Method for Whole‐Mount In Situ Hybridization of *Heterodera schachtii* Juveniles.” Parasitology Research 88: 731–733. 10.1007/s00436-002-0661-0 12122430

[mpp70346-bib-0042] Wang, S. , W. Niu , K. Wang , et al. 2025. “ *Pratylenchus coffeae* Effector PcENG2 Targets the Maize Chitinase ZmCHI5 to Promote Parasitism.” Journal of Agricultural and Food Chemistry 73: 27435–27449. 10.1021/acs.jafc.5c06425 PMC12577694 41097880

[mpp70346-bib-0043] Wang, S. , K. Wang , F. Lin , et al. 2026. “ *Pratylenchus coffeae* Effector PcENG3 Targets the Maize TCTP Protein to Facilitate Parasitism.” Crop Journal 14: 388–399.

[mpp70346-bib-0044] Waterhouse, A. M. , J. B. Procter , D. M. A. Martin , M. Clamp , and G. J. Barton . 2009. “Jalview Version 2—A Multiple Sequence Alignment Editor and Analysis Workbench.” Bioinformatics 25: 1189–1191. 10.1093/bioinformatics/btp033 PMC2672624 19151095

[mpp70346-bib-0045] Zhao, J. , K. Huang , R. Liu , et al. 2024. “The Root‐Knot Nematode Effector Mi2G02 Hijacks a Host Plant Trihelix Transcription Factor to Promote Nematode Parasitism.” Plant Communications 5: 100723. 10.1016/j.xplc.2023.100723 PMC10873892 37742073

