# Supplementary material for: MgCBP1 Synergistically Enhances MgENG2‐Mediated Cellulose Degradation to Promote Parasitism in Meloidogyne graminicola
Source: Mol Plant Pathol. 2026 Sep 17;27(9):e70346. doi: 10.1111/mpp.70346 (PMC13585997; doi:10.1111/mpp.70346)
Supplement: Supplementary file 1 [file MPP-27-e70346-s001.docx]

**Figure S1** Multiple sequence alignment of endo-β-1,4-glucanase proteins from plant-parasitic nematodes. The amino acid sequences were aligned and shaded in blue according to conservation level, with darker shades indicating higher identity. The green dashed box marks the predicted N-terminal signal peptide, while the red dashed boxes delineate the conserved glycoside hydrolase family 5 catalytic domain. Sequences included are from *Meloidogyne incognita* (MiENG1, AF323087; MiENG2, AAK21881; MiENG3, AY422836; MiENG4, AY422837), *M. javanica* (MjENG3, CAJ77137), *Heterodera glycines* (HgENG1, AF006052; HgENG2, AF006053; HgENG3, AF056048; HgENG4, AY325809), *H. avenae* (HaENG2, JN861114; HaENG3, JN861116), *H. schachtii* (HsENG1, AJ299386; HsENG2, AJ299387), *Globodera rostochiensis* (GrENG1, AF004523; GrENG2, AF004716), *Pratylenchus penetrans* (PpENG1, BAB68522; PpENG2, XCN70975; PpENG3, PV575806), and *Bursaphelenchus xylophilus* (BxENG1, BAD34543; BxENG2, BAD34544; BxENG3, BAD34545).
