# Supplementary material for: MgCBP1 Synergistically Enhances MgENG2‐Mediated Cellulose Degradation to Promote Parasitism in Meloidogyne graminicola
Source: Mol Plant Pathol. 2026 Sep 17;27(9):e70346. doi: 10.1111/mpp.70346 (PMC13585997; doi:10.1111/mpp.70346)
Supplement: Supplementary file 2 [file MPP-27-e70346-s002.docx]

**Figure S2** SDS-PAGE analysis of recombinant MBP-MgENG1 and MBP-MgENG2 expression and purification, with M indicating the protein molecular weight marker, UI the uninduced cell lysate, I the induced cell lysate, E1 and E2 the first and second elution fractions, respectively, and with the recombinant proteins MBP-MgENG1 and MBP-MgENG2 indicated by red and yellow dashed boxes, respectively.
