# Supplementary material for: MgCBP1 Synergistically Enhances MgENG2‐Mediated Cellulose Degradation to Promote Parasitism in Meloidogyne graminicola
Source: Mol Plant Pathol. 2026 Sep 17;27(9):e70346. doi: 10.1111/mpp.70346 (PMC13585997; doi:10.1111/mpp.70346)
Supplement: Supplementary file 3 [file MPP-27-e70346-s003.docx]

**Figure S3** Yeast co-transformation assay of MgCBP1 and MgENG2. The indicated bait (pGBKT7) and prey (pGADT7) plasmids were co-transformed into yeast strain Y2HGold. Transformants were selected on SD/-Leu/-Trp medium for 2 days, and protein interaction was assessed by spotting onto SD/-Ade/-His/-Leu/-Trp medium.
