# Supplementary material for: MgCBP1 Synergistically Enhances MgENG2‐Mediated Cellulose Degradation to Promote Parasitism in Meloidogyne graminicola
Source: Mol Plant Pathol. 2026 Sep 17;27(9):e70346. doi: 10.1111/mpp.70346 (PMC13585997; doi:10.1111/mpp.70346)
Supplement: Supplementary file 4 [file MPP-27-e70346-s004.docx]

**Table S1** Primers used in this study

| **Primer Name** | **Primer Sequence (5’-3’)** | **Purpose** |
| --- | --- | --- |
| Mg-CBP-1 | F：ATGAATTTTTTTAATATTTTTGTAATTGC | Amplification of MgCBP1 |
|  | R：TTAATATGGTTTACATTCCCCT |  |
| MgCBP-1-ISH | F：CAACCGCTAATCCACCTA | Template synthesis for the MgCBP1 *in Situ* hybridization probe |
|  | R:GTCCAGCAGAAAGATCAATTT |  |
| MgCBP-1-qPCR | F：CTGATGCAAACTTACCCGTTG | RT-qPCR primers for MgCBP1 |
|  | R：GAATAGATTGTCCAGCAGAAAGATC |  |
| T7-CBP1-RNAi | F1：ggatcctaatacgactcactatagggGATTCGTCTAATAATGAAGC | For the synthesis of *MgCBP1* dsRNA, the sense direction of a *MgCBP1* fragment appending T7 sequence in 5' terminus PCR amplification |
|  | R2:ggatcctaatacgactcactatagggATAGATTGTCCAGCAGAAAG |  |
| CBP1-RNAi-vitro | R1:ATAGATTGTCCAGCAGAAAG |  |
|  | F2:GATTCGTCTAATAATGAAGC |  |
| CBP1-PE | F:GAAAACCTGTACTTCCAATCCAATCCCGGGATGGAAGATTCGTCTAATAATGAAGC | prokaryotic expression vectors for MgENG1 |
|  | R:CAGGTGGCGCGCCGCGATCGCGGATCCTTAATATGGTTTACATTCCCCT |  |
| CBP1-CBMwo | F：ATCTTCATTGCGAATTGGAAAATAGAAATGCTGGTCTCG | Mutagenesis primers for the cellulose-binding domain of MgCBP1 |
|  | R:CAATTCGCAATGAAGATCAAC |  |
| MgENG1 | F:ATGGGCTTTGTAACCTTTGTAATTC | Amplification of MgENG1 and MgENG2 |
|  | R:CTAACCAGAGCAATTTAAACCATTC |  |
| MgENG2 | F:ATGAGTCTTTTAACATTTTCTATTGCC |  |
|  | R:TTATCTACCTGAGCAATTTACACC |  |
| **Primer Name** | **Primer Sequence (5’-3’)** | **Purpose** |
| MgENG1-ISH | F:AGCATTAAATAAGGGTGCTTGTCTTTTTGT | Template synthesis for MgENG1 in situ hybridization probe |
|  | R:CCAACTTGTGCTGGTGTGGTAC |  |
| MgENG2-ISH | F:GCTTGGTCAACATGGTTTTG | Template synthesis for MgENG2 in situ hybridization probe |
|  | R:GGAGTGAAGGTTCTGTCTT |  |
| MgENG1-PE | F:GAAAACCTGTACTTCCAATCCAATCCCGGGATGGGCTTTGTAACCTTTGTAATTC | prokaryotic expression vectors for MgENG1 and MgENG2 |
|  | R:CAGGTGGCGCGCCGCGATCGCCTAACCAGAGCAATTTAAACCATTC |  |
| MgENG2-PE | F:GAAAACCTGTACTTCCAATCCAATCCCGGGATGGCTTCTCCTCCTTTTGG |  |
|  | R:CAGGTGGCGCGCCGCGATCGCGGATCCTTATCTACCTGAGCAATTTACACC |  |
| MgENG2-qPCR | F:GCTTGGTCAACATGGTTTTG | RT-qPCR primers for MgENG2 |
|  | R:GGAGTGAAGGTTCTGTCTT |  |
| T7-ENG2-RNAi | F:ggatcctaatacgactcactatagggGGAGTGAAGGTTCTGTCTT | For the synthesis of *MgENG2* dsRNA, the sense direction of a *MgENG2* fragment appending T7 sequence in 5' terminus PCR amplification |
| ENG2-RNAi | R:GCTTGGTCAACATGGTTTTG |  |
| T7-ENG2-RNAi | R:ggatcctaatacgactcactatagggGCTTGGTCAACATGGTTTTG |  |
| ENG2-RNAi | F:GGAGTGAAGGTTCTGTCTT |  |
| Gfp | F:ACTTCTTCAAGTCCGCCATG | For the synthesis of *GFP* dsRNA, the sense direction of a *GFP* fragment appending T7 sequence in 5' terminus PCR amplification |
|  | R:GTGGCTGTTGTGGTTGTACT |  |
| T7-gfp | F:ggatcctaatacgactcactatagggGTGGCTGTTGTGGTTGTACT |  |
|  | R:ggatcctaatacgactcactataggGACTTCTTCAAGTCCGCCATG |  |
